# Lightweight SFRC Benefitting from a Pre-Soaking and Internal Curing Process

**DOI:** 10.3390/ma12244152

**Published:** 2019-12-11

**Authors:** Marie Hornakova, Jacek Katzer, Janusz Kobaka, Petr Konecny

**Affiliations:** 1 Faculty of Civil Engineering, VSB—Technical University of Ostrava, 708 33 Ostrava-Poruba, Czech Republic; petr.konecny@vsb.cz; 2 Faculty of Geodesy, Geospatial and Civil Engineering, University of Warmia and Mazury in Olsztyn, 10-720 Olsztyn, Poland; jacek.katzer@uwm.edu.pl; 3 Faculty of Civil Engineering, Environmental and Geodetic Sciences, Koszalin University of Technology, 75-453 Koszalin, Poland; janusz.kobaka@tu.koszalin.pl

**Keywords:** internal curing, SFRC, sustainability, recycling, waste ceramic

## Abstract

The presented research program is focused on the design of a structural lightweight fiber-reinforced concrete harnessing an internal curing process. Pre-soaked waste red ceramic fine aggregate and pre-soaked artificial clay expanded coarse aggregate were utilized for the creation of the mix. Copper-coated steel fiber was added to the mix by volume in amounts of 0.0%, 0.5%, 1.0%, and 1.5%. Test specimens in forms of cubes, cylinders, and beams were tested to specify the concrete characteristics. Such properties as consistency, compressive strength, splitting tensile strength, static and dynamic modulus of elasticity, flexural characteristics, and shear strength were of special interest. The achieved concrete can be classified as LC12/13. A strength class, according to *fib* Model Code, was also assigned to the concretes in question. The proposed method of preparation of concrete mix using only pre-soaked aggregate (with no extra water) proved to be feasible.

## 1. Introduction

In recent decades, there has been a growing interest in the field of sustainability. In terms of civil engineering, two of the many problems within this field are the massive amounts of demolished brickwork and concrete and the manufacturing of new concrete and bricks, which cause the reduction of natural resources. Therefore, the recycling and reusing waste or unsuitable building materials as a total or partial replacement of traditional concrete ingredients is of highest interest. The ceramic industry produces large amounts of waste each year. The specific characteristics of red ceramic utilized as aggregate could improve the properties of concrete through harnessing the process of internal curing. Lightweight concrete is characterized by density (ρ) significantly lower than the density of ordinary concrete (circa 2400 kg/m^3^). Usually, the density threshold for concrete to be classified as lightweight is defined as ρ < 1800 kg/m^3^. The reduction of concrete density can be achieved in different ways: by using porous lightweight aggregates, by introducing a large volume of voids within the concrete mass (e.g., foamed concrete [1], which has the unique advantage of low exploitation of natural resources and raw materials), and by omitting the fine aggregate from the mixture [2]. In this project, two lightweight aggregates were used: one artificial and one waste, namely, expanded clay coarse aggregate (ECCA) and waste red ceramic fine aggregate (WRCFA), which were harnessed to achieve low density of hardened concrete. In this research program, only fine waste ceramic aggregate was used. The limitations and opportunities of using coarse waste ceramic aggregate for concrete production were discussed by authors in multiple previous publications [3,4,5,6]. The aim of the research program is the creation of a concrete mixture which contains the already-mentioned pre-soaked aggregates, limited amount of cement (about 300 kg per 1 m^3^) and different amount of steel fiber without using additional water. Determined material characteristics of the fresh mix and hardened concrete would be influenced solely by water trapped in both porous aggregates and the process of internal curing [3]. Using only pre-soaked aggregate and no extra water would significantly simplify the production process of lightweight concrete. The novelty of the conducted research program is fully associated with this issue.

The suitability of lightweight concrete for construction applications is conducted by the needed properties: density, strength, and thermal conductivity. Nevertheless, other properties, such as workability, water absorption, drying shrinkage and moisture movement should also be considered. The porous structure of lightweight aggregates causes high and rapid water absorption during preparation of the fresh mix. Thus, if dry porous aggregate is used for the creation of a mixture, it rapidly absorbs free water, and the workability of the fresh concrete mix is significantly affected [2]. Although the saturation of aggregates can initially cause the increase of the concrete density, it improves the concrete’s long-term maturation by slowly releasing water from pores. The internal curing process significantly influences the speed and quality of cement hydration. It leads to the enhancement of mechanical properties of hardened concrete and reduces autogenous shrinkage [3].

An important aspect of the research program is to deepen the knowledge about the usage of waste red ceramic aggregate. The recycling and usage of waste material in the construction field have been discussed by many research groups [3,4,5,6,7,8,9,10,11], but in practice, it is still not very common to reuse the old material as a part of new concrete. However, to encourage extensive further usage, there is need to establish a standardization for waste aggregate concrete.

## 2. Used Materials

**Figure 1:**
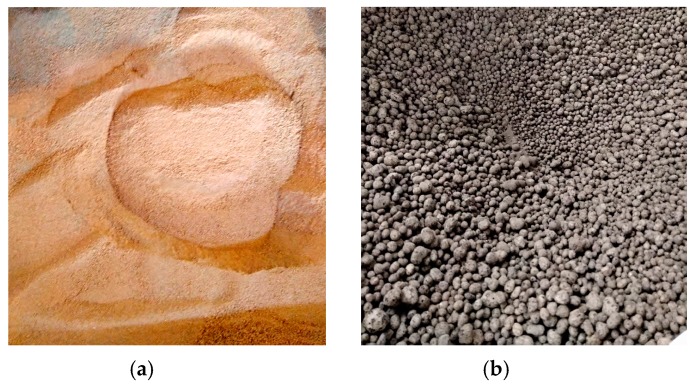
(**a**) Waste red ceramic fine aggregate (WRCFA) and (**b**) expanded clay coarse aggregate (ECCA).

WRCFA (Figure 1a) was solely used as fine particles of the mixture. This aggregate was procured through crushing and grinding red ceramic debris. The debris was sourced from a local producer of airbricks. In the European Union, construction elements which did not pass quality-control tests are not allowed to leave the production facility. Therefore, they are usually destroyed on site, creating huge stockpiles of clean debris. Small and medium producers of bricks often struggle with the problem of recycling or utilization of piling up ceramic debris. For the purpose of the research program, the red ceramic debris was sorted by size of the particles. Only fine particles (diameter *d* ≤ 2 mm) were used. Harnessing coarse particles of the waste aggregate for the creation of different concrete mixes was described in multiple previous publications [5,7]. One should note that such aggregate is clean and does not contain leftover mortar.

Commercially available artificial lightweight aggregate was used as coarse aggregate. The expanded clay coarse aggregates (ECCA, Figure 1b) are produced on a large scale by mixing clay and water into paste. The clay dries and expands in the rotary kiln at temperatures of 1100–1200 °C. The expanding happens due to the generation of gases which become enmeshed in the mass. The final particle is expanded porous clay with a hard shell. The pores are mostly interconnected [12].

**Figure 2:**
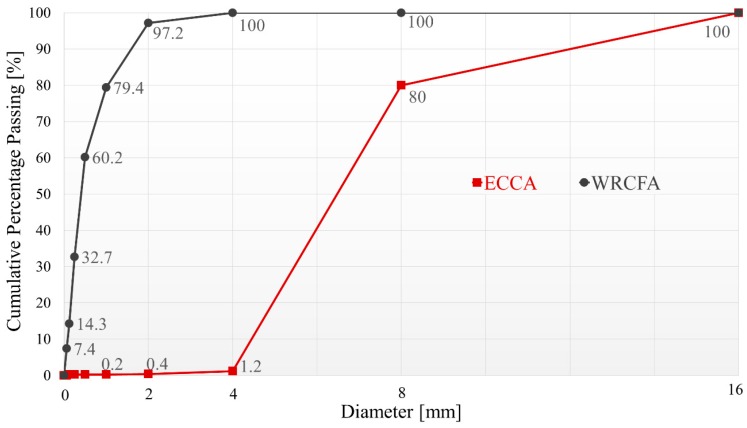
Grading curves of the aggregates.

Properties of both aggregates were tested before the very creation of the concrete mixes. Sieve analysis was conducted according to EN 933-2:1996 [13] using rectangular sieves of the following apertures: 0.63, 0.125, 0.25, 0.5, 1.0, 2.0, 4.0, and 8.0 mm. Achieved grading curves are presented in Figure 2. Values of different fineness moduli and median diameter [14] calculated for both aggregates are presented in Table 1. Apart from geometrical properties, absorptivity, loose and compacted bulk density were tested according to the EN 1097-6 [15] and EN 1097-3 [16], respectively. Measured values are given in Table 1. Portland Cement I 42.5 N-NA [17] was used as a binder to prepare all the mixes in question. Only tap water [18] was utilized throughout the research program.

**Figure 3:**
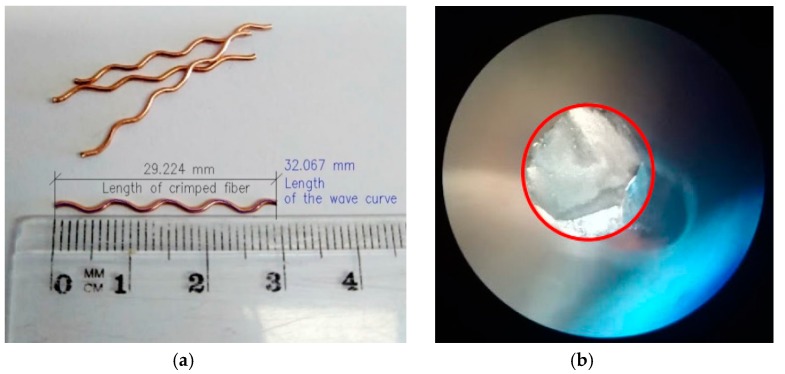
(**a**) Copper-coated crimped steel fiber and (**b**) its cross-section in a microscopic view.

Copper-coated crimped steel fibers (Figure 3a) were utilized as reinforcement. This fiber was chosen after the review of literature. Crimped fiber is one of the four most popular fiber types in the concrete industry [19]. Nevertheless, the vast majority of the literature discussing steel-fiber-reinforced concrete (SFRC) is dedicated to concretes reinforced by hooked steel fiber. Authors decided to use a different type of steel fiber than a hooked one to broaden the technological knowledge. Synthetic fibers were not considered for utilization during the research program due to their limited mechanical characteristics. Geometric properties of this commercially available fiber were thoroughly tested. Properties of the fiber are presented in Table 2.

The fiber cross-section is circular (Figure 3b), so the perimeter *ψ* and cross-sectional area *A* were calculated. The straightened length *L* of every fiber was calculated based on the mean value of the ratio of the wave curve length and the crimped fiber length of 5 fibers. The fiber intrinsic efficiency ratio was determined according to Equation (1) [20].

(1) $$FIER=\frac{A\psi }{L}$$

**Figure 4:**
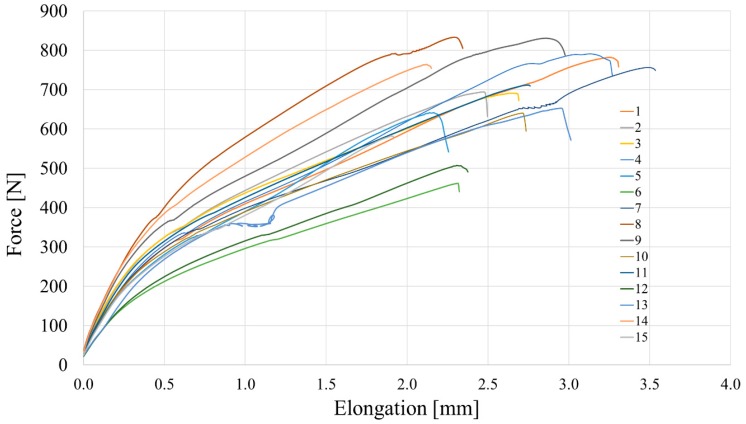
Behavior of fiber during the tensile test.

The tensile strength of the fiber was tested on 15 randomly chosen fibers. the behavior of fiber during the tensile test is presented in Figure 4. This characteristic is important for the analysis of flexural test results of SFRC beams. Crimped steel fibers are prone to much larger elongation during the tensile test than hooked steel fiber due to the initial geometric shape. Based on the mean values of failure forces and cross-section areas of fibers, the ultimate tensile strength 1.7 ± 0.3 GPa was determined [21].

## 3. Design of the Concrete Mix

The main purpose of the mix proportioning is to create a formula or a recipe fulfilling a certain requirement (whether mechanical or technological). The requirements for the mixture in question were very specific and at the same time quite unconventional: using only pre-soaked lightweight aggregates (no additional water), good workability of the fresh mix, minimum voids on the surface of samples (closed structure).

**Figure 5:**
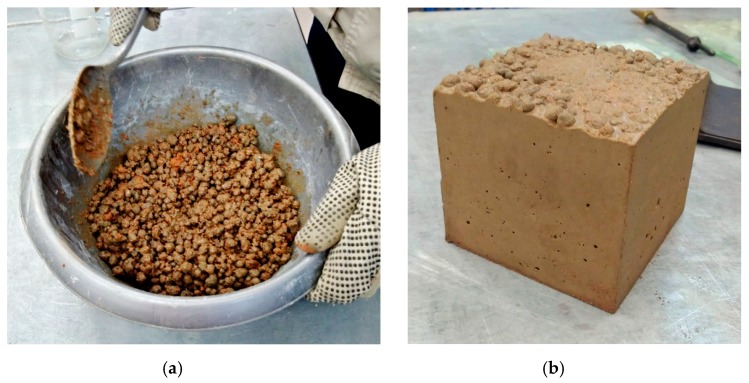
(**a**) Trial mixture and (**b**) first cast specimen.

There are two methods for designing lightweight structural concrete—a weight method and a volumetric method, but it is recommended to use proportions previously established for a similar concrete mixture [22,23]. Thus, the trial mixture was created based on the authors’ experience. During this attempt, only a small amount of water was added to achieve a good workability of the mix (Figure 5a). The trial recipe proved to be sufficient for the purposes of the research program because the surface of the specimen was closed as it was requested (Figure 5b). The final mixture created in much larger quantity did not require the addition of any water. The workability of the mix was satisfactory. The proportions of the mixture for a cubic meter are given in Table 3. The fibers were added by volume (*V_f_*) of concrete 0.0%, 0.5%, 1.0%, and 1.5%. The volumes of added fiber represent the most common additions of steel fiber to concrete. Adding less than 0.5% of fiber does not influence concrete in a noticeable way. On the other hand, using more than 1.5% of fiber would require utilization of an admixture or special mixing and compaction techniques. 

All mixes were prepared in a rotary drum mixer. The mixing procedure lasted 3 min and consisted of 1 min of mixing the aggregate and cement, 1 min of slowly adding the fibers while continuing mixing, and 1 min of mixing all ingredients. After casting the fresh mix into molds, the compaction was performed externally, utilizing a vibrating table for 30 s. Adopted mixing and casting procedures resulted in random 3D spacing of added fiber. The procedures were successfully utilized in previous research programs focused on SFRC based both on natural and waste aggregates.

## 4. Conducted Tests and Results

Immediately after the mixing, the slump test (according to EN 12350-2:2009 [24]) was conducted. Classification of achieved consistency was performed using workability classes of ordinary concrete [25,26]. The consistency of cast mixes is presented in Table 4.

The specimens were cast in a form of cubes (for compressive and splitting tensile strength), cylinders (for static and dynamic modulus of elasticity) and beams (for flexural characteristics and shear strength). For the first 24 h, the specimens were kept in their molds tightly covered with polyethylene sheets. Then, the specimens were removed from the molds and put into a water tank. The specimens were cured in the water tank for the next 27 days at a temperature of +21 °C. After the 28-day curing process was finished, all specimens were measured and weighted. The determined density of hardened concretes is presented in Table 5. All created concretes can be classified as lightweight [26] because their density is significantly less than 1800 kg/m^3^.

**Figure 6:**
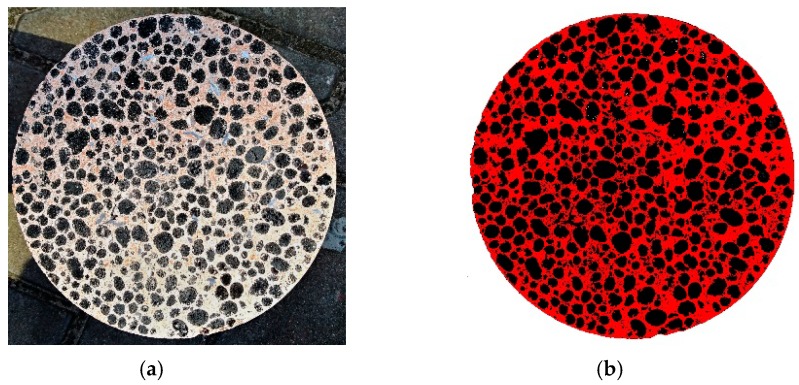
(**a**) Cross-section of the cylindrical specimen in real and (**b**) artificial colours.

With the help of the open-source graphic software [27], a simple assessment of the ratio of mortar (containing WRCFA and cement) and ECCA was conducted. The graphic program is able to determine a number of pixels of a given color. The picture of a cross-section of a cylinder specimen was modified in the program to get artificial black and red colors. Subsequently, the pixels were counted (Figure 6). The total amount of pixels is equal to 3.038 × 10^6^, the number of pixels representing ECCA in the cross-section is equal to 1.003 × 10^6^, and the amount of pixels representing mortar is equal to 2.035 × 10^6^, which closely corresponds to the ratio of the mix ingredients given in Table 3.

### 4.1. Compressive Strength

To test the compressive strength *f_c_* [28] three cube specimens (100 × 100 × 100 mm) were used. The specimens were placed into the compression-testing machine and continuously loaded at a constant speed (0.6 ± 0.2 MPa/s) to the point of ultimate failure. The result of the testing is the force *F* at the failure point in kN. The compression strength is given by Equation (2).

(2) $$f_{c}=\frac{F}{A_{c}}$$

where: *F*—the maximum force; *A_c_*—the area.

**Figure 7:**
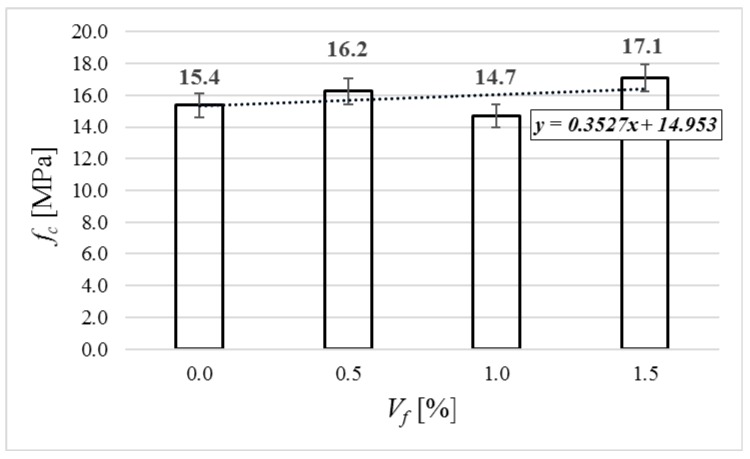
Compressive strength of tested specimens.

Figure 7 shows the average results of the compressive strength. The compressive strength ranges from 14.7 to 17.1 MPa. In comparison to the concrete with no fiber, the changes of the compressive strength are reasonably small (from −4.5% to +11.0%). It is a commonly known phenomenon that fiber addition does not influence basic compressive strength in any significant way [20]. Small losses of compressive strength in comparison to concrete matrix are also common [29]. All tested concretes may be classified as LC 12/13 according to [26].

### 4.2. Splitting Tensile Strength

To test the splitting tensile strength *f_t,spl_* [30], three cube specimens (100 × 100 × 100 mm) were used. The specimens were placed into the compression-testing machine and continuously loaded at a constant speed (0.6 ± 0.2 MPa/s) to the point of ultimate failure. The splitting tensile strength is given by Equation (3).

(3) $$f_{t,spl}=\frac{2\cdot F}{\pi \cdot L\cdot d}$$

where: *F*—the maximum force; *L*—contact length of the specimen; *d*—transverse dimension of the specimen.

**Figure 8:**
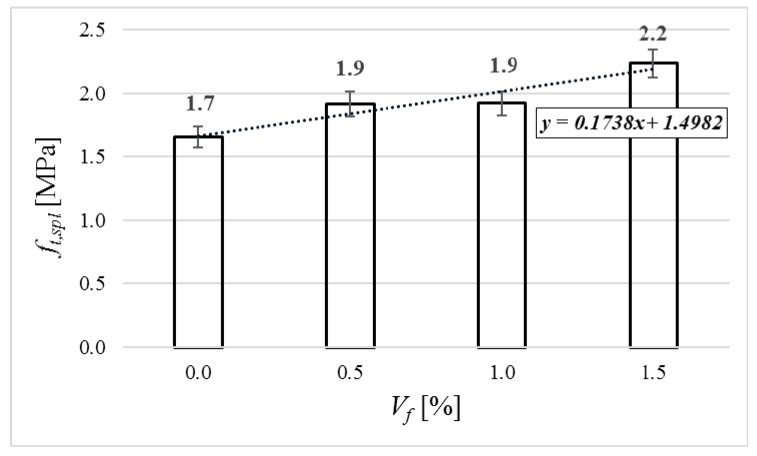
Splitting tensile strength of tested specimens.

The average results of the splitting tensile strength are given in Figure 8. The value of the splitting tensile strength raised up with the volume of added fiber. For fiber addition of 0.5% and 1.0%, the value was larger by 11.8% in comparison to the concrete with no fiber. In the case of fiber addition of 1.5%, the value was larger by 29.4% in comparison to the concrete with no fiber.

### 4.3. Static Modulus of Elasticity

**Figure 9:**
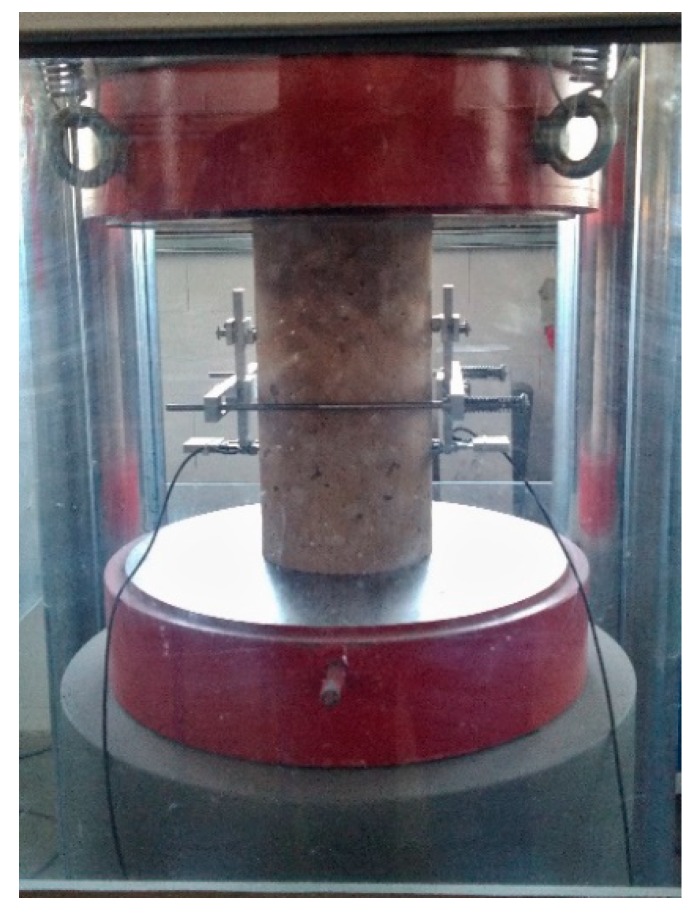
Testing setup of the static modulus of elasticity.

Testing the static modulus of elasticity [31] was executed on three cylindrical specimens with the diameter of 150 mm and height of 300 mm (see Figure 9). The modulus was calculated using the following equation:

(4) $$E_{c}=\frac{\sigma_{a}-\sigma_{b}}{\varepsilon_{a}-\varepsilon_{b}}$$

where: *σ_a_*—upper load stress; *σ_b_*—lower load stress; ε_a_—mean value of upper load stress strain; ε_b_—mean value of lower load stress strain.

**Figure 10:**
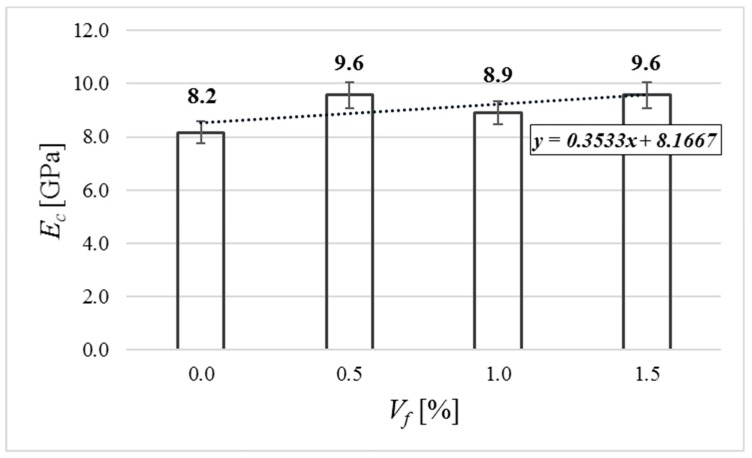
Static modulus of elasticity of tested specimens.

The average results of static modulus of elasticity are presented in Figure 10. It can be observed that the value increases with added fiber. In the case of concrete with added fiber in volumes of 0.5% and 1.5%, the static modulus of elasticity increased by 17.1%, and in the case of *V_f_* = 1.0%, the static modulus of elasticity increased by 9%.

### 4.4. Dynamic Modulus of Elasticity

The testing of the dynamic modulus of elasticity (*E_cu_*) was conducted using an ultrasound method. A commercially available ultrasonic testing instrument, usually harnessed for non-destructive testing of concrete strength, was utilized [32]. The velocity of the ultrasound wave was determined (see Equation (5)). Then, the dynamic modulus of elasticity was calculated using Equation (6):

(5) $$v_{L}=\frac{L}{T}$$

where: *L*—length of the specimen, *T*—time of the single pulse pass.

(6) $$E_{cu}=\rho \cdot v_{L}^{2}$$

where: *ρ*—the density of the material.

**Figure 11:**
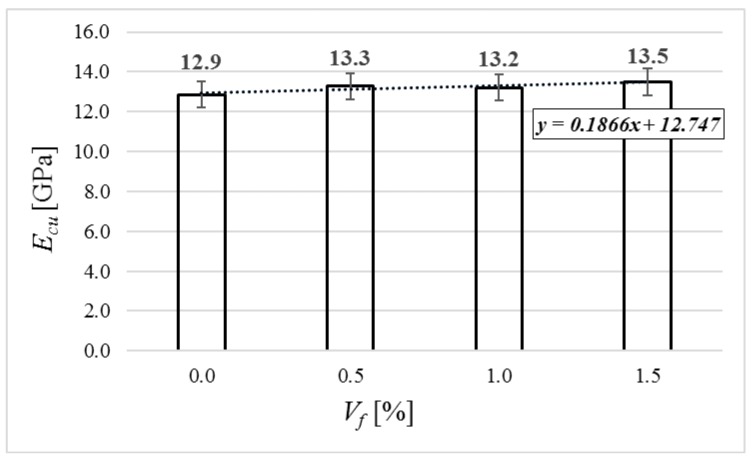
Dynamic modulus of elasticity of tested specimens.

The average result of dynamic modulus of every mixture is shown in Figure 11. The values increased up to 4.6% in comparison to concrete with no fiber.

### 4.5. Flexural Characteristics

**Figure 12:**
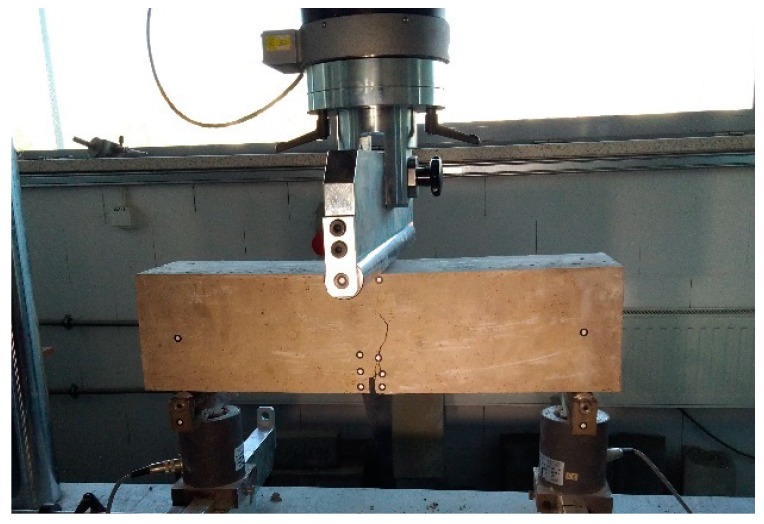
Three-point bending test of a notched beam.

The flexural characteristics of steel-fiber-reinforced concrete (SFRC) is usually assessed by residual flexural tensile strength values that are calculated based on load-crack mouth opening displacement curve or load–deflection curve [33]. A three-point bending test was conducted on three beams of every mixture as shown in Figure 12. The crack-mouth opening displacement (CMOD) was measured using a digital image correlation system. The applied system proved to be efficient in testing fiber-reinforced concrete and was thoroughly described in a previous publication [34]. The values of deflections and loads were registered by the strength-testing machine. The ultimate flexural tensile strength of the specimens without fiber was calculated using Equation (7) [35]. The limit of proportionality (LOP) of SFRC specimens was calculated using Equation (8) [33].

(7) $$f_{LOP}=\frac{3\cdot F\cdot l}{2\cdot b\cdot h_{sp}^{2}}$$

where: *F*—the maximum load, *l*—the span, *b*—the width of the specimen, *h_sp_*—the distance between the tip of the notch and the top of the specimen.

(8) $$f_{LOP}=\frac{3\cdot F_{L}\cdot l}{2\cdot b\cdot h_{sp}^{2}}$$

where: *F_L_*—the load corresponding to the LOP.

Residual flexural strength was calculated using Equation (9).

(9) $$f_{R,j}=\frac{3\cdot F_{j}\cdot l}{2\cdot b\cdot h_{sp}^{2}}$$

where *F_j_*—the load corresponding with *CMOD* = *CMOD_j_* or *δ* = *δ_j_* (*j* = 1,2,3,4).

**Figure 13:**
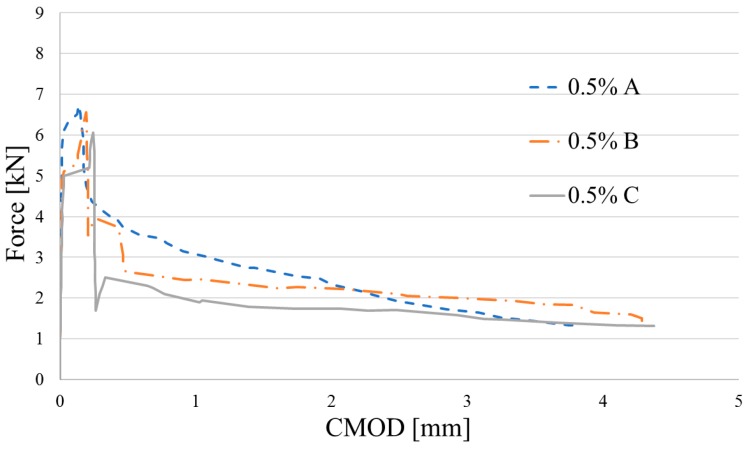
Flexural characteristics of concrete reinforced by *V_f_* = 0.5% of fiber.

**Figure 14:**
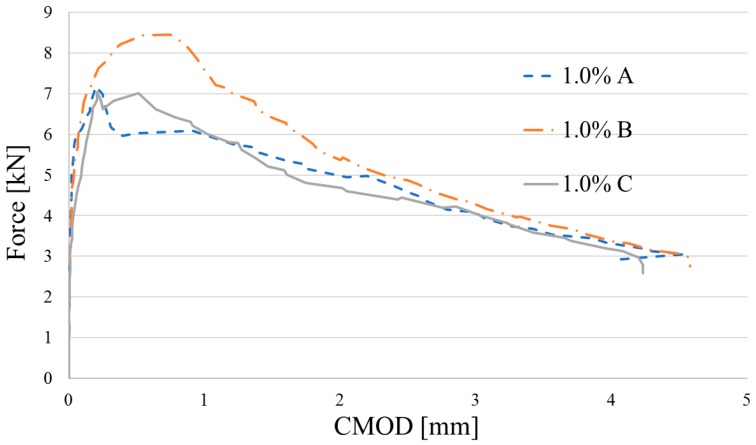
Flexural characteristics of concrete reinforced by *V_f_* = 1.0% of fiber.

**Figure 15:**
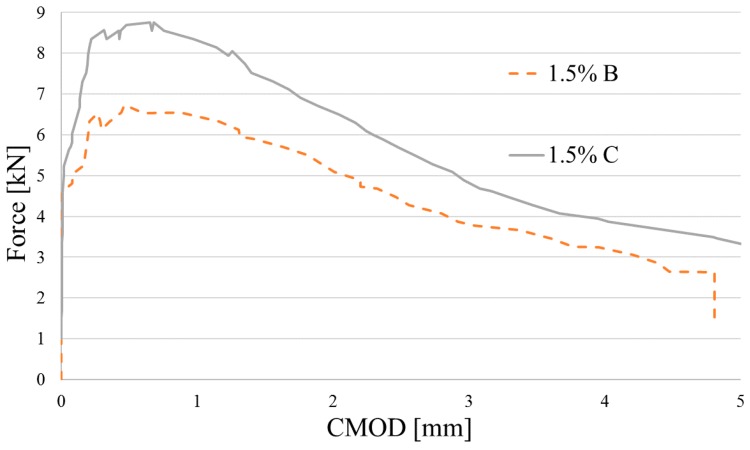
Flexural characteristics of concrete reinforced by *V_f_* = 1.5% of fiber.

**Figure 16:**
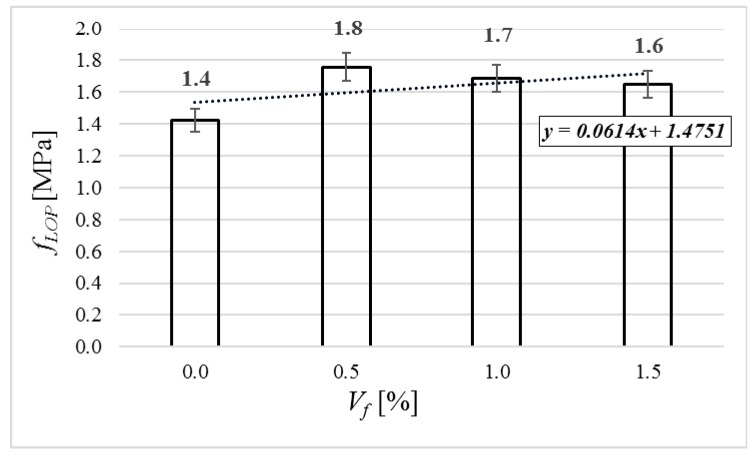
Limit of proportionality of tested specimens.

Flexural characteristics of tested beams are shown in Figure 13, Figure 14 and Figure 15. Because of technical difficulties, the results of the first beam with 1.5% of fibers are unfortunately lost. The limits of proportionality are shown in Figure 16. The highest value of LOP can be observed for concrete with 0.5% of fibers. Then, the value slowly decreases.

The properties computed based on the bending test and the classification of the SFRC are given in Table 6. The value *f_R1_* and the *f_R3_/f_R1_* ratio (see *fib* Model Code 2010 [36]) was used for the classification. This classification represents the most common cases of hardening and softening of SFRC (where *a* stands for “pure” softening and *d* stands for “pure” hardening). Fiber reinforcement can be used also as a substitution of conventional reinforcement to avoid brittleness if the following assumptions are fulfilled [25].

(10) $$f_{R3}/f_{R1}\geq 0.5$$

(11) $$f_{R1}/f_{LOP}\geq 0.4$$

### 4.6. Shear Strength

Shear strength was tested on the half-beam leftovers after conducted flexural tests. The specimens were loaded using shear apparatus continuously, without impact. The values of shear strength were calculated by using Equation (12) [37].

(12) $$f_{shear}=\frac{F}{2\cdot A}$$

where: *F*—the maximum force, *A*—an area of the cross-section of the specimen. 

**Figure 17:**
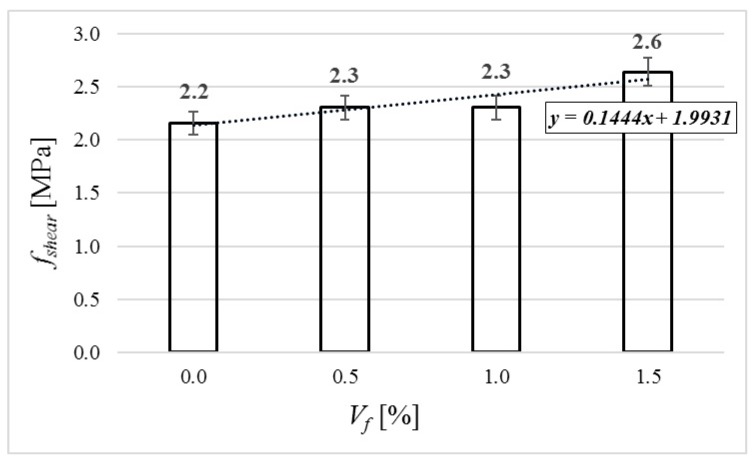
Shear strength of tested specimens.

The average results of the shear strength are shown in Figure 17. The value increases with the volume of the fiber: in the cases of 0.5% and 1.0% of fibers by 7%, and in the case of 1.5% of fibers by 22%.

## 5. Discussion

All planned mixes were feasible to create and cast. They were workable and characterized by density and compressive strength ranging from 1366.7 to 1484.9 kg/m^3^ and from 14.7 to 17.1 MPa, respectively. These are common values achieved by commercially available lightweight concretes. The uniqueness of the concretes in question is associated with the method of their creation and achieved mechanical characteristics. Creating a lightweight concrete mix is much more difficult than creating an ordinary concrete mix, especially in situ. Most technological problems are usually associated with high water absorption by lightweight aggregate. The aggregate soaks up significant amounts of water, influencing the consistency and workability of the fresh mix. This phenomenon forces producers to use excessive amounts of water or superplasticizer. Both methods have their weaknesses, and they do not guarantee good quality of either the fresh mix or, subsequently, the hardened concrete. In the authors’ opinion, using fully pre-soaked lightweight aggregate makes the process of the creation of lightweight concrete easier and much more predictable. The proposed procedure is feasible to execute even in very basic in situ conditions. Additionally, the created composite benefits from the internal curing process. Due to limited mechanical characteristics, lightweight concretes are commonly used for secondary construction applications. Reinforcing the lightweight concrete in question through the addition of steel fiber enhanced some of its mechanical properties. On the other hand, fiber addition did not influence in a statistically significant way the compressive strength and dynamic modulus of elasticity of the lightweight concrete. This phenomenon is well known, tested, and described in the case of steel-fiber-reinforced concretes based on ordinary aggregate. A similar behavior of fiber-reinforced concretes was noted by multiple researchers [38,39] when using synthetic fibers in lightweight concretes.

Fiber addition influenced splitting tensile strength, static modulus of elasticity, and shear strength by 30%, 17%, and 18%, respectively (for *V_f_* = 1.5%). Due to fiber addition, the lightweight concrete gained quasi-plastic flexural characteristics. According to fib Model Code 2010 [36], the tested fiber concretes are 0.5a, 2.0a, and 2.0a strength class for *V_f_* = 0.5%, *V_f_* = 1.0%, and *V_f_* = 1.5%, respectively. In all three cases, the substitution of traditional reinforcement is enabled. The conducted research program proved that pre-soaking lightweight aggregate (especially WRCFA) enables the reasonably easy production of lightweight steel-fiber-reinforced concrete. This non-conventional concrete is in need of further lab and application tests. It creates a realistic opportunity for harnessing huge volumes of waste red ceramics.

## 6. Conclusions

The research program allows drawing the following conclusions:

- It is feasible to create concrete using only pre-soaked porous aggregates and no extra water;
- The proposed method of the creation of lightweight concretes with no extra water is easy to execute both in laboratory and in situ;
- Achieved density and basic compressive strength enable created concretes to be classified using strength classes according to EN 206-01;
- Achieved flexural characteristics enabled using *fib* Model Code 2010 [36] strength classes of SFRC;
- All cast SFRC are enabled for reinforcement substitution;
- The mechanical properties of cast SFRC enable using them for the chosen structural applications;
- Additional tests associated with the durability and thermal conductivity of concretes in question should be conducted.

## Figures and Tables

**Table 1:** Properties of used aggregates.

Aggregate	Fineness Modulus by			Median Diameter	Bulk Density		Water
	Hummel	Abrams	Kuczynski		Loose	Compacted	Absorptivity
	*m_H_* [-]	*m_A_* [-]	*m_K_* [-]	*d_m_* [mm]	[kg/m^3^]	[kg/m^3^]	[%]
ECCA	915.57	5.98	0.50	6.48	318.8	345.8	36
WRCFA	587.83	2.16	0.62	0.41	1183.8	1489.2	46

**Table 2:** Properties of copper-coated steel fiber.

Nominal		FIER	Aspect Ratio	Density
Length [mm]	Diameter [mm]	[-]	[-]	[kg/m^3^]
30.8	0.73	169.4	42.3	7800

**Table 3:** Mixture proportions of a cubic meter of mixture.

	WRCFA [kg/m^3^]	ECCA [kg/m^3^]	Cement [kg/m^3^]	Fiber [kg/m^3^]	
dry	378.38	247.07	320.49	*V_f_* = 0.5%	39.0
soaked	700.71	386.05	-	*V_f_* = 1.0%	78.0
absorbed water	322.33	138.98	-	*V_f_* = 1.5%	117.0

**Table 4:** Consistency of cast mixes.

*V_f_* [%]	Slump [mm]	Consistency Class (EN 12350-2)
0.0%	80.0	S2
0.5%	70.0	S2
1.0%	70.0	S2
1.5%	32.5	S1

**Table 5:** Density of specimens.

Specimens		Density [kg/m^3^]			
Shape	Size [mm]	*V_f_* = 0.0%	*V_f_* = 0.5%	*V_f_* = 1.0%	*V_f_* = 1.5%
Cubes	100 × 100 × 100	1356.99	1405.06	1434.94	1480.00
Cylinders	Φ150 × 300	1370.86	1413.00	1444.63	1500.21
Beams	150 × 150 × 600	1372.03	1444.15	1471.31	1474.45
Mean value		1366.63	1420.74	1450.29	1484.89

**Table 6:** Flexural characteristics.

Properties	*V_f_* [%]			
	0.0	0.5	1.0	1.5
*f_LOP_* [MPa]	1.42 *	1.76	1.69	1.65
**Residual Strength**				
*f_R1_* [MPa]	n/a	0.83	2.28	2.46
*f_R2_* [MPa]	n/a	0.72	1.82	2.12
*f_R3_* [MPa]	n/a	0.61	1.48	1.61
*f_R4_* [MPa]	n/a	0.50	1.17	1.25
**Strength Class and Reinforcement Substitution According to *fib* Model Code**				
*f_R3_/f_R1_*	n/a	0.66	0.65	0.65
*f_R1_/f_LOP_*	n/a	0.53	1.35	1.49
Class according to *fib* Model Code	n/a	0.5a	2.0a	2.0a
Reinforcement substitution	n/a	enabled	enabled	enabled

* In the case of concrete with no fiber, this value represents ultimate flexural tensile strength.
